# Oil body biogenesis and biotechnology in legume seeds

**DOI:** 10.1007/s00299-017-2201-5

**Published:** 2017-09-02

**Authors:** Youhong Song, Xin-Ding Wang, Ray J. Rose

**Affiliations:** 1 School of Agronomy, Anhui Agricultural University, Hefei, 230036 People’s Republic of China; 2 School of Environmental and Life Sciences, The University of Newcastle, Callaghan, NSW 2308 Australia

**Keywords:** Oil bodies, Oil body biogenesis, Lipid droplets, *Medicago truncatula*, Soybean, Legume biotechnology, Legume oil production

## Abstract

The seeds of many legume species including soybean, *Pongamia pinnata* and the model legume *Medicago truncatula* store considerable oil, apart from protein, in their cotyledons. However, as a group, legume storage strategies are quite variable and provide opportunities for better understanding of carbon partitioning into different storage products. Legumes with their ability to fix nitrogen can also increase the sustainability of agricultural systems. This review integrates the cell biology, biochemistry and molecular biology of oil body biogenesis before considering biotechnology strategies to enhance oil body biosynthesis. Cellular aspects of packaging triacylglycerol (TAG) into oil bodies are emphasized. Enhancing seed oil content has successfully focused on the up-regulation of the TAG biosynthesis pathways using overexpression of enzymes such as diacylglycerol acyltransferase1 and transcription factors such as WRINKLE1 and LEAFY COTYLEDON1. While these strategies are central, decreasing carbon flow into other storage products and maximizing the packaging of oil bodies into the cytoplasm are other strategies that need further examination. Overall there is much potential for integrating carbon partitioning, up-regulation of fatty acid and TAG synthesis and oil body packaging, for enhancing oil levels. In addition to the potential for integrated strategies to improving oil yields, the capacity to modify fatty acid composition and use of oil bodies as platforms for the production of recombinant proteins in seed of transgenic legumes provide other opportunities for legume biotechnology.

## Introduction

**Fig. 1:**
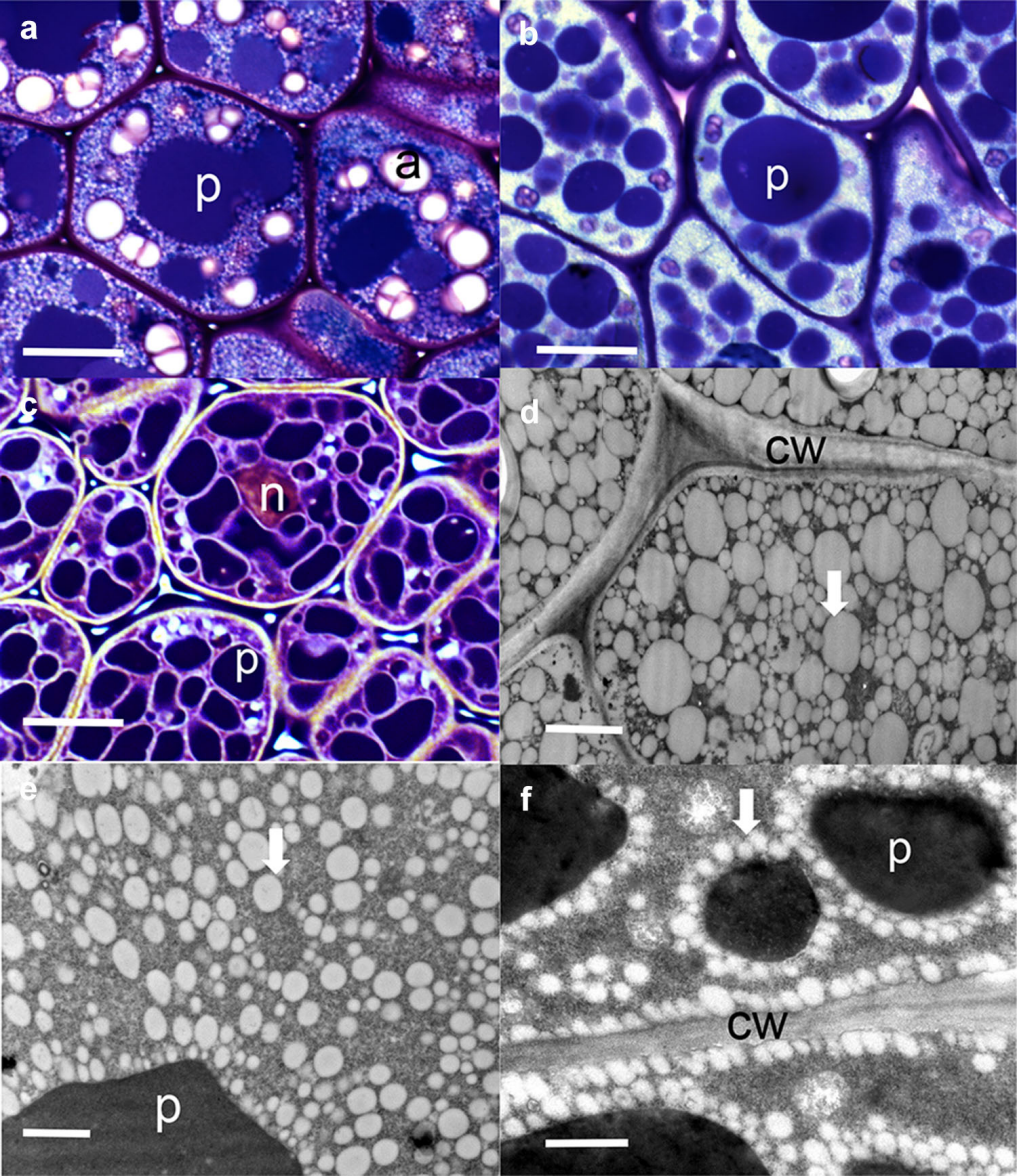
Oil bodies in three legumes: *Pongamia*, soybean and *Medicago*. Light microscope images of cotyledon storage cells of *Pongamia* (**a**), soybean (**b**) and *M. truncatula* (**c**). In *Pongamia* and soybean, oil bodies are *white dots* filling the cytoplasm, while in *M. truncatula* they are *white borders* ringing the protein bodies. Electron microscope images of oil bodies of *Pongamia* (**d**), soybean (**e**) and *M. truncatula* (**f**). *a* amyloplast, *n* nucleus, *cw* cell wall, *p* protein body, *arrows* point to individual oil bodies. *Bars* **a**–**c** 10 µm and **d**–**f** 1 µm

Legume seeds store proteins, lipids and starch required for energy and growth upon germination. The proportion of these storage components varies according to species (Table 1; Fig. 1). The seed can thus be harvested to serve human nutrition, stock feed, biofuels and industrial application (Duranti and Gius 1997; Djemel et al. 2005; Gallardo et al. 2008). Legume seed can accumulate considerable oil aside from protein, and the oil content is largely dependent on legume species (Table 1). For example, soybean seed contains 20% oil (Clemente and Cahoon 2009). *Pongamia pinnata* (*Pongamia*) seed commonly stores 35% oil, which offers a potential source for biofuel use (Scott et al. 2008). Low oil is usually associated with high starch, but not necessarily (Table 1). As the world population is growing rapidly there is increasing pressure on food security, and seed oil production is part of this. In addition, biodiesel demand for future energy also relies on greater seed oil production. Therefore, improvement of seed oil production will be important in addressing these challenges (Roesler et al. 2016).

**Table 1:** The approximate composition (% dry wt) of common legume species

Legume species	Protein (%)	Oil (%)	Starch (%)	References
*Medicago truncatula*	35	9	2	Djemel et al. (2005)
*Glycine max*	40	20	2	Guillon and Champ (2002)
*Pongamia pinnata*	20	35	**7**	Scott et al. (2008), Bala et al. (2011)
*Arachis hypogea*	26	52	4	Pickett (1950)
*Cicer arietinum*	23	6	44	Huang et al. (2007), Rachwa-Rosiak et al. (2015)
*Lupinus luteus, angustifolius and albus*	38	8	2	Guillon and Champ (2002)
*Pisum sativum*	27	3	40	Guillon and Champ (2002)
*Vicia faba*	30	3	45	Guillon and Champ (2002)
*Phaseolus vulgaris*	23	2	19	Mohamed et al. (1995)
*Lotus japonicus*	43	7	1	Dam et al. (2009)

Legumes are second to cereals in agricultural importance, based on area harvested and total production (Gepts et al. 2005). Grain legumes provide about one-third of dietary protein nitrogen and vegetable oil for human consumption (Graham and Vance 2003). Intercropping and rotation of grain legumes with cereals or other non-leguminous crops can increase nitrogen use efficiency and enhance yield (Rose 2008; Siddique et al. 2012). It has been estimated that crop legumes contribute 21 Mt (megatonne, 10^6^ tonnes, tonne is the metric ton) of N_2_ annually by legume–rhizobia symbioses (Herridge et al. 2008) and return 5–7 Mt of N_2_ to soil saving US$8–12 billion (Foyer et al. 2016). Further, synthetic nitrogen fertilizers are a major source of nitrous oxide emissions (Davidson 2009; Foyer et al. 2016). Nitrous oxide is the fourth most important anthropogenic greenhouse gas. It is also a reactant in the destruction of atmospheric ozone (Davidson 2009). In addition, it is argued that legumes have been underutilized, with a negative impact on human health and sustainable food production (Foyer et al. 2016).

In this review we first integrate the cell biology, biochemistry and molecular biology of oil body biogenesis. We then examine biotechnological strategies for modification of oil content. Enhancing seed oil content has to date particularly focused on the up-regulation of the oil biosynthesis pathways. While this strategy is important, decreasing carbon flow into other storage products and maximizing the packaging of oil bodies into the cytoplasm need further examination. The capacity to modify fatty acid composition and the use of oil bodies as platforms for the production of recombinant proteins in seed of transgenic legumes are also considered.

## Oil bodies in legumes

Seed oil is stored as oil bodies (also known as lipid droplets) which are subcellular structures in the cytoplasm (Fig. 1). Oil bodies are generally considered to be circular to ovoid, with diameter varying between species, but within the range of 0.5–2.5 µm (Huang 1992; Tzen et al. 1993; Wang et al. 2012). However, as can be seen for *Pongamia* and soybean (Fig. 1) some oil body diameters can be 2–3 times larger than 2.5 µm. Oil bodies are filled with triacylglycerols (TAGs) enclosed by a monolayer of phospholipid (PL) embedded with integral membrane oleosin proteins (Huang 1992; Tzen et al. 1993; Tzen 2012). There are different isoforms of oleosin and two minor integral proteins, caleosin and steroleosin (Chen et al. 1999; Tzen 2012). The structure of oil bodies allow them to be maintained as small discrete structures (Fig. 1). In *Medicago truncatula* oil bodies are aligned around the protein bodies (Fig. 1f) and adjacent to the plasma membrane. In legumes with more lipids the oil bodies pack into the cytoplasm with different patterns of oil body sizes (Fig. 1d, e). It is possible that the arrangement of oil bodies adjacent to the plasma membrane may have a cellular protective effect during seed desiccation. Knockdown of oleosin in soybean, which leads to the formation of giant oil bodies, results in few, if any, viable cotyledon cells after hydration and germination (Schmidt and Herman 2008).

The oil body can be detected as early as the heart stage in *Brassica napus* (He and Wu 2009). The different oleosin isoforms, caleosin and steroleosin accumulate sequentially throughout seed development (Gallardo et al. 2016). Fatty acid accumulation starts from 8 days after pollination, corresponding to the heart stage in *M. truncatula* (Wang et al. 2012). While this review focuses on seed oil bodies, oil bodies can be present in many different tissues and organs (Gallardo et al. 2016).

## Oil body biogenesis

The pathway from photosynthate to the final oil bodies is composed of three major parts: fatty acid biosynthesis, TAG assembly and oil body formation. As a consequence, oil body formation in developing seed is regulated at multiple steps.

### Fatty acid synthesis

**Fig. 2:**
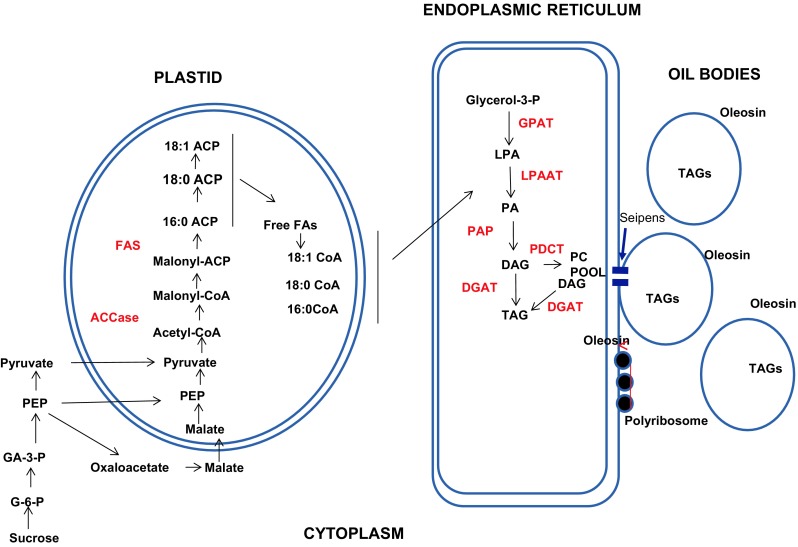
Oil body biogenesis summary diagram. Biosynthesis of fatty acids and oil bodies. Information from Ohlrogge and Browse (1995), Chapman and Ohlrogge (2012), Tzen (2012), Bates et al. (2013), Bates (2016), Xu and Shanklin (2016), Pyc et al. (2017). *PEP* phosphoenolpyruvate, *GA-3-P* glyceraldehyde-3-phosphate, *G6-P* glucose-6-phosphate, *ACCase* acetyl-CoA carboxylase, *FAS* fatty acid synthase, *ACP* acyl carrier protein, *FAs* fatty acids, *GPAT* glycerol-3-phosphate acyltransferase, *LPA* lysophosphatidic acid, *LPAAT* lysophosphatidic acid acyltransferase, *PA* phosphatidic acid, *PAP* phosphatidic acid phosphatase, *DAG* diacylglycerol, *DGAT* diacylglycerol acyltransferase, *PC* phosphatidylcholine, *PDCT* PC:DAG cholinephosphotransferase, *TAG* triacylglycerol. The accumulation of TAGs occurs between the two unit membranes of the ER. This would place at least some reactions shown diagrammatically, within the ER membrane

Fatty acids are synthesized de novo in seed plastids using the sucrose imported from photosynthesis (Fig. 2). The sucrose is first cleaved into glucose and fructose, followed by processing into pyruvate via glycolysis. While pyruvate may enter the plastid directly from the cytoplasm by a passive mechanism, or unknown translocator, there are other sources of pyruvate in soybean plastids (Hajduch et al. 2011; Gerrard Wheeler et al. 2016). Phosphoenol pyruvate (PEP) can be imported into the plastid via a PEP translocator and pyruvate formed via a plastid pyruvate kinase. Likely more important in soybean (Hajduch et al. 2011) is the production of malate from oxaloacetate derived from PEP. Malate is imported into the plastid via a dicarboxylic translocator and converted to pyruvate by a NADP malic enzyme (Hajduch et al. 2011; Gerrard Wheeler et al. 2016). In the plastid, pyruvate dehydrogenase converts pyruvate to CO_2_, which is recycled, and acetyl-CoA which is utilized for fatty acid biosynthesis (Fig. 2). The enzyme acetyl-CoA carboxylase (ACCase) is generally considered to catalyse the first reaction of the fatty acid biosynthetic pathway: the formation of malonyl-CoA from acetyl-CoA. Malonyl-CoA is used for the production of 16:0 ACP, 18:0 ACP and 18:1 ACP by the fatty acid (FA) synthase (Chapman and Ohlrogge 2012). Two carbon fragments are added to the growing FA chain, which is covalently attached to the acyl carrier protein (ACP). Free FAs are produced from 16:0 ACP, 18:0 ACP and 18:1 ACP by two acyl-ACP thioesterases (Ohlrogge and Browse 1995; Chapman and Ohlrogge 2012). The FAs are activated to CoA on the chloroplast outer envelope and 16:0 CoA, 18:0 CoA and 18:1 CoA are transferred to the endoplasmic reticulum (ER) for TAG assembly. The majority of commonly grown oil crops accumulate mainly C16 or C18 saturated and unsaturated fatty acids in their storage lipids.

### TAG assembly

Lipids are stored in the seed in the form of TAGs, in which fatty acids are linked by ester bonds with the three available hydroxyl groups of glycerol (Ohlrogge and Browse 1995; Bates et al. 2013; Fig. 2). Fatty acids are transferred from acyl-CoA to the glycerol-3-phosphate backbone at the sn-1 position by glycerol-3-phosphate acyltransferase (GPAT) and the sn-2 position by the lysophosphatidic acid acyltransferase (LPAAT), yielding the central metabolite phosphatidic acid (PA). Phosphatidic acid is then dephosphorylated to diacylglycerol (DAG) and a third fatty acid is transferred to the free sn-3 position of DAG by diacylglycerol acyltransferase (DGAT). DAG can also be converted to phosphatidylcholine (PC) and flux through a PC pool, and then made available for TAG biosynthesis (Bates et al. 2013; Xu and Shanklin 2016). This last fatty acid transfer step for TAG biosynthesis in the sn-3 position, carried out by DGATs, is specific to TAG biosynthesis. There are two major distinctive DGATs (DGAT1 and DGAT2) located in the ER membrane (Bates et al. 2013). Their relative activity is species dependent. There is also an acyl-CoA-independent reaction catalysed by phospholipid:diacylglycerol acyltransferase (PDAT1), not shown in Fig. 2, which uses phospholipids as an acyl donor and DAG as an acyl acceptor to produce TAGs and lysophospholipids (Xu and Shanklin 2016).

### Oil body assembly

Free TAGs undergo further packaging into oil bodies. As outlined above, the mature oil bodies in the cytosol are filled with a TAG matrix surrounded by a PL monolayer membrane embedded with the abundant oleosin protein of ~15–30 kDa (Chapman et al. 2012) and the minor caleosin and steroleosin proteins. There are two abundant oleosin isomers that may form heterodimers or heteromultimers on the surface of the oil bodies (Huang 1996). The current understanding is that oleosins are co-translationally inserted into the ER and associate with the TAGs which accumulate between the two phospholipid leaflets (Hsieh and Huang 2004; Shimada and Hara-Nishimura 2010; Chapman et al. 2012; Tzen 2012). The TAGs surrounded by the phospholipid monolayer, with the embedded proteins, eventually bud off to form the oil body (Figs. 1, 2). The models are supported by ultrastructure studies (Herman 2008), linking oleosin biosynthesis to the ER (Sarmiento et al. 1997) and the thermodynamics involved (Huang 1992). Because of their hydrophobicity, the TAGs synthesized in the ER can be sequestered between the two PL layers (Huang 1996). Models for the organization of the integral oil body proteins (oleosin, as well as caleosin and steroleosin) on the surface of the oil body can be found in the review by Tzen (2012) on integral proteins in plant oil bodies. Oleosin has an N-terminal amphipathic domain, a central lipophilic anchoring domain and a C-terminal amphipathic domain (Huang 1992; Tzen et al.^1993^; Tzen 2012). Around 20% of amino acid residues are immersed in the PL layer, 30% are located in the TAG matrix, and the remaining 50% are exposed to the cytosol (Tzen et al. 1993). Oleosin stabilizes the oil bodies and prevents coalescence of oil bodies by electronegative repulsion and steric hindrance (Tzen et al. 1992; Tzen and Huang 1998). The structural role of oleosins allows the oil bodies to be maintained as relatively small discrete organelles (Tzen 2012) that can be packed into the cytoplasm (Fig. 1). Even with the cytoplasmic compression during dehydration in late maturation, where oil bodies are forced into contact with each other, the organelles resist coalescence and remain as small individual bodies due to oleosins (Murphy 2001). The large surface area per unit of TAG also facilitates lipolysis during germination (Hsieh and Huang 2004).

Extensive phylogenetic studies have identified five oleosin lineages (Huang and Huang 2015). These are P (primitive; in algae, mosses and ferns), U (in all land plants), SL (seed low—molecular weight, in seeds of gymnosperms and angiosperms), SH (seed high molecular weight in seeds of angiosperms) and T (tapetum, in tapeta of Brassicaceae). Legumes have U, SL and SH oleosins (Huang and Huang 2016). In soybean, with 13 oleosin genes, 2 SL and 2 SH oleosin genes produce 90% of the oleosin. A role for different oleosin isoforms in oil body dynamics has been demonstrated using Arabidopsis mutants where not only oil body size, but also spatial distribution is influenced (Miquel et al. 2014). Miquel et al. also show that fusion of smaller oil bodies is a contributor to oil body growth. It seems plausible that the different oil body sizes and distribution are influenced by the way different oleosins are expressed. This could contribute to the differences seen in Fig. 1 between *Pongamia* and soybean oil body size distribution, and is worthy of further investigation. Another outstanding question in oil body assembly is how the composition difference between the unit phospholipid membrane and the cytosolic leaflet of the ER is generated (Miquel et al. 2014).

The biogenesis of oil bodies can occur in the absence of oleosins in other tissues and organs (Chapman et al. 2012). In the mesocarp of avocado and olive there are ‘oil bodies’ that likely lack oleosin and are subsequently very large, from 10 to 20 μm in diameter; while oil bodies in the seed contain oleosin and are 0.5–2.0 μm in diameter (Ross et al. 1993). Importantly, it is the small oil bodies in the seed that undergo desiccation and are then mobilized for germination (Murphy 2012). It does suggest that the oleosins are important in seed oil bodies to meet the storage, desiccation and germination requirements of the seed. In a more recent study on avocado mesocarp cells (Huang and Huang 2016), oleosin transcripts of a special phylogenic lineage (designated M) were observed in the mesocarp cells. Very small oil bodies (<0.5 μm) on the periphery of the cell in addition to the very large ones (>5.0 μm) were identified. The oleosin was mainly associated with the small oil bodies as determined by immuno-confocal laser scanning microscopy. Very large oil bodies may lack oleosins and be stabilized by the lipid-associated proteins LDAP1 and LDAP2 (Horn et al. 2013) which are similar to small rubber particle proteins. In green algae, oleosins have been detected but are not major proteins (Huang et al. 2013). The 28 kDa hydrophobic protein MLDP (‘Major Lipid Droplet Protein’) is the most abundant protein associated with oil bodies in green algae and shares no primary sequences with oleosin; MLDP, however, appears to influence oil body size and prevent fusion (Goold et al. 2015). In Chlamydomonas when MLDP synthesis is inhibited, oil body size increases (Goold et al. 2015). In addition to oleosins, there are a number of other proteins associated with plant oil bodies (Gallardo et al. 2016). It seems likely that this includes peripheral proteins associated with the surface structures of oil bodies for oil body mobilization during germination (Feussner et al. 2001; Jolivet et al. 2013).

While oleosin has a major influence on oil body size and distribution and maintains the integrity of the oil body in desiccation, seipen is another protein that is important in determining the number and size of oil bodies. Seipens in plants were discovered as homologues of animal and yeast seipens (Cai et al. 2015). Three seipens were found in Arabidopsis and there are three homologues in *M. truncatula* (Cai et al. 2015). Transient expression of the Arabidopsis genes in *Nicotiana benthamiana* showed that SEIPIN1 caused the accumulation of large oil bodies, while expression of *SEIPIN2* and *SEIPIN3* accumulated small oil bodies. *SEIPEN1* has its highest expression in developing seeds (Cai et al. 2015). GFP-tagged SEIPENS localized to oil body-forming sites on the ER of *N. benthamiana* leaves, with the ER identified by the ER marker protein CFP-HDEL (Cai et al. 2015). The morphology of the ER after the co-labelling with GFP-tagged SEIPENS and the CFP-HDEL suggested that SEIPENS are involved in organizing sub-domains of the ER devoted to TAG synthesis and oil body assembly (Fig. 2).

## Transcription factors and the regulation of oil body biogenesis

The transcriptional regulation of oil body biogenesis in legumes has not been as extensively investigated compared to Arabidopsis with its 36% seed oil storage (Li et al. 2006), availability of mutants and ease of transgenic analysis.

The transcription factor most directly linked to fatty acid biosynthesis is WRINKLED1 (WRI1), discovered from the isolation of the Arabidopsis *wri1* mutant (Focks and Benning 1998). WRINKLED1 regulates genes encoding most of the key enzymes of the later stages of glycolysis and fatty acid biosynthesis (Focks and Benning 1998; Cernac and Benning 2004; Kim et al. 2016). Homologues of *WRI1* are widely spread, from Arabidopsis to oil palm (Ma et al. 2013). Homologues are present in legumes (Ma et al. 2013) but have not been the subject of experimental work. It seems likely that in higher plants *WRI1*, or closely related genes, is the important regulator of fatty acid biosynthesis. WRI1 is a member of the AP2/ERBP transcription factor family (Cernac and Benning 2004) and binds to the AW box sequence [5′-[CnTnG](n)7[CG]-3′] of promoters of the target glycolysis and fatty acid biosynthesis genes (Maeo et al. 2009). Further investigations by Kim et al. (2016) have identified MEDIATOR15 (MED15), a subunit of the Mediator complex, as an interacting partner of WRI1. The work suggests that WRI1 targets the promoters of glycolysis-related and fatty acid biosynthesis genes and then recruits the mediator complex and RNA polymerase II, via MED15, to activate transcription. Another transcriptional regulator (Shi et al. 2012) implicated in the regulation of oil biosynthesis is GLABRA2 (GL2). This is discussed in the “Biotechnology—increasing oil production” section below.

Oil body biogenesis has been linked to the transcriptional regulators of embryo maturation, given that the production of seed storage reserves goes hand in hand with the maturation of the embryo (Kurdyukov et al. 2014). Four ‘master’ transcription regulators have been identified in Arabidopsis as controllers of seed maturation (Meinke et al. 1994; Gutierrez et al. 2007) together with LEAFY COTYLEDON1-like (L1L, Kwong et al. 2003). The four master regulators are LEAFY COTYLEDON1 (LEC1) (West et al. 1994; Lotan et al. 1998; Casson and Lindsey 2006), LEAFY COTYLEDON2 (LEC2) (Stone et al. 2001), FUSCA3 (FUS3) (Bäumlein et al. 1994; Parcy et al. 1997) and ABSCISIC ACID-INSENSITIVE3 (ABI3; Nambara et al. 1995; Parcy et al. 1997). LEC2, FUS3 and ABI3 belong to the B3 family of transcription factors, with B3 DNA-binding domains (Stone et al. 2001). LEC1 belongs to the CBF family of transcription factors and encodes a CCAAT box-binding HAP3 subunit (Lee et al. 2003). LIL is similar to LEC1 but encodes a different HAP3 subunit (Kwong et al. 2003).

A number of studies have directly implicated the master transcription factors in the regulation of oil biosynthesis. Mu et al. (2008) using *LEC1*-overexpressing Arabidopsis plants under the control of an estradiol-inducible promoter found that a number of enzyme-coding genes in the plastid fatty acid synthesis pathway were up-regulated, including three subunits of ACCase. Genes involved in glycolysis and lipid accumulation were also up-regulated; and oil bodies formed in vegetative tissues. LEC1 function was found to be partially dependent on ABI3, FUS3 and WRI1 (Mu et al. 2008). LEC1 and LEC2 act upstream of ABI3 and FUS3 (To et al. 2006; Wang et al. 2007). FUS3 (Wang et al. 2007) but not ABI3 (Mu et al. 2008) was able to directly stimulate oil biosynthesis genes when overexpressed in Arabidopsis (Wang et al. 2007). In *B. napus* both *LEC1* and *LIL* when overexpressed act similar to *LEC1* overexpression in Arabidopsis (Mu et al. 2008), consistent with similar functions of LEC1 and LIL (Kwong et al. 2003).

Using transgenic Arabidopsis plants with an inducible *LEC2*, it was shown that LEC2 is somewhat similar to LEC1 in its action. *LEC2* activation triggered the accumulation of oil- and seed-specific mRNAs and induced *LEC1*, *FUS3* and *ABI3* (Santos Mendoza et al. 2005).

LEC2 and LEC1 (Mu et al. 2008) also control the expression of the AP2/ERFP transcription factor WRI1 which is essential for oil accumulation (Focks and Benning 1998; Cernac and Benning 2004; Baud et al. 2007). Though WRI1 acts downstream of LEC1 and LEC2, and they appear to regulate WRI1, binding to the WRI1 promoter by LEC1 and LEC2 has not been demonstrated (Marchive et al. 2014). It has recently been found that the transcription factor MYB89 is able to directly repress WRI1 (Li et al. 2017). WRI1 and related transcription factors have a key role in the controlled production of fatty acids (Focks and Benning 1998; Cernac and Benning 2004; Baud et al. 2007; To et al. 2012; Marchive et al. 2014).

In Legumes, the sequencing of a number of genomes, *Lotus japonicus* (Sato et al. 2008), soybean (Schmutz et al. 2010), *M. truncatula* (Young et al. 2011), chickpea (Varshney et al. 2013), lupin (Yang et al. 2013) and common bean (Schmutz et al. 2014), has enabled identification of orthologues of the legume transcription factors shown to regulate oil body biogenesis in Arabidopsis. In *M. truncatula* there is a range of proteomic (Gallardo et al. 2007; Thompson et al. 2009) and gene expression data (Gallardo et al. 2007; Udvardi et al. 2007; Verdier et al. 2008; Kurdyukov et al. 2014; Nolan et al. 2014) in relation to the master regulators of oil body biogenesis. There have not been overexpression studies that have been carried out in other species.

## Biotechnology—increasing oil production

In addition to up-regulation of lipid biosynthesis, to maximize oil body production it is necessary to consider the partitioning of carbon into oil and packaging of the lipid into oil bodies of suitable size and cellular distribution.

### Modifying carbon partitioning

Examination of Table 1 indicates the variable nature of carbon partitioning in legumes and offers the potential for investigating partitioning into different storage products. A number of legumes (e.g. soybean, *Pongamia*) are rich in oil and protein while others are rich in starch and protein (e.g. chickpea, faba bean), and in some cases protein is quite dominant (e.g. lupin). Where oil is relatively low (Table 1) there can be a trade-off with increased starch (e.g. chickpea) or fibre (e.g. lupin). A hierarchical comparative genomics analysis of ten legume genomes to produce multiple alignments of homologous genes is a new resource for legume biology, which can aid comparative studies (Wang et al. 2017). More than 850 oil synthesis-related genes were located in the peanut genomes and 1528 in soybean (Wang et al. 2017).

There has been experimentation on the effects of reducing starch on oil content. In peas, Weigelt et al. (2009) used RNAi to repress ADP-glucose pyrophosphorylase (AGP) to reduce starch. Reduced starch was associated with increased protein, lipid and sucrose. In mature seed, lipid increased from 40 to 58 mg g^−1^ and total N increased from 2.9 to 4.3%. There was, however, a yield penalty with seed weight reduced from 345 to 302 mg. There were some negative stress signalling effects but there were responses to circumvent these (Weigelt et al. 2009). In Arabidopsis, using RNAi against AGP was combined with overexpression of the WRI1 transcription factor (Sanjaya et al. 2011). This resulted in less starch and more hexoses and 5.8-fold more oil in vegetative tissues compared with reduced AGP alone or overexpressed *WRI1* alone.

In considering carbon partitioning in seed, cell wall storage polysaccharides (mannans, xyloglucans and galactans) are important components (Buckeridge et al. 2000) which have received little attention in legumes. In legumes, mucilage (a galactomannan) is located in the endosperm cell wall (Edwards et al. 1999; Naoumkina et al. 2008). The main function of the endosperm cell walls appears to be storage, with the yield of galactomannan reaching more than 30% of the seed dry weight in many legume species (Buckeridge 2010). Some years ago, in a study based on 300 legume species, it was shown that there was an inverse relationship between legume mucilage and oil plus protein (Tookey and Jones 1965). We have also shown this inverse relationship between *Medicago orbicularis* with low oil (Tonnet and Snudden 1974) and high endosperm mucilage compared to *M. truncatula* (unpublished data).

In Arabidopsis, seed coat mucilage which is released from the cell wall on hydration forms a gel-like capsule surrounding the seed. The mucilage may mediate seed dispersal through adhesion or possibly facilitate seed hydration (Western 2012). Carbon partitioning into increased oil production can also be obtained in Arabidopsis by reducing the expression of the transcription factor GLABRA2 (GL2). Mutants of the GL2 transcription factor (Shi et al. 2012) have reduced mucilage on the seed surface and increased oil production. While mucilage is not present in the seed coat of legumes (e.g. *M. truncatula*; Verdier et al. 2013), as pointed out above, it is present in the remaining seed endosperm. Mucilage and other storage polysaccharides are potentially available to contribute to oil production, perhaps by down-regulating *GL2* homologues.

Interestingly, in the study by Roesler et al. (2016) in soybean using improved DGAT variants, a 3.5 percentage point increase of oil and protein was accompanied by a 1.9 percentage point reduction of soluble sugars. Preliminary analysis also indicated a reduction of cell wall polysaccharides and likely the oligosaccharides raffinose and stachyose.

Investigations of the interaction between three of the master regulators of seed maturation LEC2, ABI3 and FUS3 in Arabidopsis provided some information on the partitioning between storage products using single, double and triple mutants and ectopic overexpression (Roscoe et al. 2015). It was concluded that LEC2 influences lipid and protein storage to a similar extent, while ABI3 increased protein relative to lipid and the converse for FUS3.

### Metabolic engineering of enzymes involved in TAG biosynthesis

A number of enzyme-coding genes involved in fatty acid synthesis, glycerol synthesis and ultimately TAGs when overexpressed increase oil production (Weselake et al. 2009). These include genes involved in fatty acid synthesis (e.g. homomeric ACCase, Roesler et al. 1997), synthesis of the glycerol backbone (e.g. glycerol-3-phosphate dehydrogenase, Vigeolas et al. 2007), GPAT (Jain et al. 2000) and LPAAT (Zou et al. 1997) involved, respectively, in the first and second acyl-CoA-dependent acylation of the glycerol backbone. Most prominent has been DGAT (Fig. 2) which catalyses the final acyl-CoA-dependent acylation, transferring an acyl group from acyl-CoA to diacylglycerol. This last fatty acid transfer step for TAG biosynthesis in the sn-3 position, carried out by DGATs, is specific to TAG biosynthesis.

DGAT investigations have resulted in field trials demonstrating increased oil production in both canola (*B. napus*) and soybean. In Arabidopsis overexpression of seed-specific DGAT cDNA increased the oil content from 34 to 46% (Jako et al. 2001). In canola (*B. napus*) *DGAT1* genes from Arabidopsis or *B. napus* increased oil content from 2.5 to 7% on an absolute dry matter basis, under field conditions (Taylor et al. 2009). In soybean a DGAT1 variant increased the percentage of seed oil by 3 percentage points (approximately from 19 to 22%) based on the T2 and T3 transgenic generations in field trials (Roesler et al. 2016). This increase in seed oil was an improvement on previous studies where the increase was 1.5 percentage points using a DGAT2 from the fungus *Umbelopsis ramanniana* optimized for expression in soybean (Lardizabal et al. 2008) or a DGAT1 from *Sesamum indicum* (Wang et al. 2014). The study by Roesler et al. (2016) used amino acid substitutions in soybean DGAT1. The amino acid substitutions were based on the amino acid composition of DGAT1 from the American hazelnut shrub *Corylus americana*. There are suggestions that DGAT1 overexpression is less sensitive to environmental stress and warrants further study in this context, given the sensitivity of oil production to abiotic stress (Singer et al. 2016).

In *Camelina sativa* it has been shown that DAG supply can be enhanced by co-expressing two Arabidopsis phospholipase D_ζ_ genes (*AtPLD*_*ζ*_ *1* and *AtPLD*_*ζ*_ *2*) which convert PC to DAG (Yang et al. 2017). This results in a 2–3% increase of TAGs in total seed biomass. Bates (2016) has argued that an enhanced understanding of the control of acyl flux through the lipid metabolic network is required for the best approaches to genetic engineering of oilseeds.

Liu et al. (2015) have been able to show that simultaneous overexpression of multiple TAG biosynthetic genes in a high-oil canola cultivar can increase oil content more than overexpression of a single gene. The simultaneously overexpressed genes were *GPDH*, *GPAT*, *LPAAT* and *DGAT*.

### Transcription factors

The overexpression of the master regulators of seed maturation provide another approach to increasing seed oil content, particularly LEC1 and LEC2 which are able to regulate FUS3 and ABI3 as well as WRI1 (Weselake et al. 2009). As indicated above, when *LEC1* is overexpressed in Arabidopsis it is able to regulate many genes involved in oil body biogenesis (Mu et al. 2008). This also leads to increases in the level of major fatty acid species and increases in fatty acid biosynthesis also occur when *B. napus LEC1 and L1L* are overexpressed in *Arabidopsis.* Tan et al. 2011 have shown that seed-specific expression of *BnLEC1* and *BnL1L* using truncated *napin A* promoters in transgenic *B. napus* increased seed oil by 2–20%. Recent studies with *BnLEC1* overexpressed constitutively in *B. napus* led to an increased seed oil content of 7–16% (Elahi et al. 2016).

Ectopic expression of *LEC2* in Arabidopsis vegetative leaves can also induce storage oil in leaves (Santos Mendoza et al. 2005; Stone et al. 2008). Similar results were obtained with transgenic expression of *FUS3* which quickly induced fatty acid synthesis in transgenic seedlings and mesophyll protoplasts (Wang et al. 2007). Clearly master TFs are important in approaches to enhance oil body synthesis. Both LEC1 and LEC2 act upstream of FUS3 and ABI3 (Wang et al. 2007; Weselake et al. 2009). It seems based on existing information that *LEC1*or *LEC1L* are the master regulators of choice for improved oil biosynthesis (Mu et al. 2008; Weselake et al. 2009; Tan et al. 2011; Elahi et al. 2016).

LEC1 and LEC2 both target the WRI1 transcription factor which has been shown to more directly target fatty acid synthesis. Therefore, it is not surprising that WRI1 overexpression enhances oil biosynthesis in Arabidopsis (Cernac and Benning 2004) and maize (Pouvreau et al. 2011) and is the current focus for enhancing oil biosynthesis (Marchive et al. 2014; Horn and Benning 2016). *WRI1* has been a gene of choice in combination with other genes. Vanhercke et al. (2013) have demonstrated what they call a ‘push’ and ‘pull’ approach to obtain increased plant oil levels in *N. benthamiana* leaves. The ‘push’ involved using *WRI1* to increase fatty acid levels and the ‘pull’ to increase TAG assembly using *DGAT1*. Co-expression of these two genes resulted in a synergistic effect. Given the success of *DGAT1* overexpression in increasing soybean levels (Roesler et al. 2016) the additional use of *WRI1 i*s attractive.

### Promoting TAG packaging into oil bodies

The size of oil bodies is related to the ratio of oleosin to TAGs. A high ratio of oil to oleosin produces large oil bodies, while small oil bodies have a low ratio of oil to oleosin (Shimada and Hara-Nishimura 2010). This offers the potential to modify oil production by optimizing the oleosin level. A deficiency of oleosin results in an inhibition of oil content (Siloto et al. 2006). Down-regulation of oleosin in Arabidopsis leads to increased oil body size, due to coalescence, and decreased oil production (Siloto et al. 2006). A similar relationship between oleosin and final oil body size has been shown for soybean (Schmidt and Herman 2008). Suppression of soybean oleosin caused the formation of giant oil bodies, observed at the onset of dormancy, but final oil content was not reported. In canola, higher oil content is associated with higher oleosin (Hu et al. 2009) and large oil bodies are characteristic of the lower oil content. Rice seed oil can be increased by overexpression of soybean oleosin and there are more numerous and smaller oil bodies (Liu et al. 2013). Overexpression of *DGAT1* and a stabilized cysteine [Cys]-oleosin in Arabidopsis leaves enabled the accumulation of TAG to 2.1% dry weight, 44-fold higher than wild type (Winichayakul et al. 2013). Transient co-expression of *WRI1* and *OLEOSIN1* in *N. benthamiana* leaves also increased TAG production (Zhai et al. 2017).

SEIPEN1 can also been used to influence oil content and oil body size. An increased seed oil content of up to 10% in Arabidopsis has been obtained by ectopic expression of *SEIPEN1* and was accompanied by an increase in oil body size compared to wild type (Cai et al. 2015). An integral ER protein in animals called FAT STORAGE-INDUCING TRANSMEMBRANE PROTEIN 2 (FIT2) when co-expressed with LEC2 and DGAT2 increased oil content. This suggests FIT2 is present in plant cells and shows the value of ‘push’ (LEC2), ‘pull’ (DGAT2) and ‘protect’ (FIT2) strategies (Pyc et al. 2017).

Oleosins also provide other opportunities for other biotechnological strategies for oil bodies. As discussed by Horn and Benning (2016), it may be possible to use oleosins to interact with other proteins that could act as catalysts for reactions to broaden the engineering capacity of plants. It could be a useful way of fortifying seeds with various proteins for health or to enhance seed protein yields. Yi et al. (2015) used oleosin translational fusion technology (Van Rooijen and Moloney 1995a, b) to produce human growth factor 9 fused to oleosin in Arabidopsis cells.

### Modifying fatty acid composition

In addition to oil production, specific fatty acids are best suited for specific purposes such as human health needs, industrialized products and biofuels (Clemente and Cahoon 2009; Haslam et al. 2013). There has been important progress in this area. In soybean, lines with high oleic acid levels have been produced by reducing expression of fatty acid desaturase 2 genes, which catalyse the conversion of the monounsaturated oleic acid to the polyunsaturated linoleic acid, using RNAi (Buhr et al. 2002; Wagner et al. 2011) or by mutations produced by TALENS (Haun et al. 2014; Demorest et al. 2016). The monounsaturated oleic acid is considered better for cardiovascular health than the polyunsaturated linoleic acid. Reduced linoleic acid improves oxidative stability without the production of trans-fatty acids (Clemente and Cahoon 2009). The omega-3 long-chain polyunsaturated fatty acids (LC-PUFAs) such as eicosapentaenoic acid (EPA) and docosahexaenoic (DHA) have been linked to reduced risk of cardiovascular disease and improved aspects of cognition and mental health (Haslam et al. 2013). These fatty acids are not known to be produced in higher plants and are present in fish oil (Haslam et al. 2013). Marine fish oil is considered to be insufficient to meet increasing demand for LC-PUFAs (Haslam et al. 2013; Horn and Benning 2016). In *C. sativa* up to 12% EPA and 14% DHA of total fatty acids have been obtained when co-synthesized and 31% when EPA alone was targeted (Ruiz-Lopez et al. 2014; Horn and Benning 2016). This was achieved by redirecting endogenous 18:3 through a series of desaturases and elongases from microalgae and fungi. The construct designed to accumulate EPA and DHA in the transgenics involved seven genes (Ruiz-Lopez et al. 2014). In soybean EPA levels of almost 20% of the total seed fatty acids have been produced (Clemente and Cahoon 2009). Long-chain *w3* fatty acids and long-chain *w6* fatty acids have been produced in transgenic Arabidopsis (Qi et al. 2004) and soybean (Sato et al. 2004). Similar to modifying fatty acids to improve health outcomes fatty acid modification can be used to optimize oil for industrial and biofuel industries. High-oleic and low-palmitic oils are more oxidatively stable than commodity soybean oils which results in reduced NO_x_ emissions (Kinney and Clemente 2005; Santos et al. 2013).

## Conclusions and future prospects

There has been successful utilization of the understanding of fatty acid and TAG biosynthesis to increase seed oil in legumes using overexpression of key genes. There are opportunities to build on this using the so-called ‘push’, ‘pull’ and ‘protect’ approach (Pyc et al. 2017). Current genomics in legumes makes it possible to readily locate orthologues based on studies in other species. The optimization of oil body assembly for maximum oil is not completely clear, e.g. see the different oil body size patterns between *Pongamia*, soybean and *M. truncatula* oil bodies (Fig. 1). However, both oleosins and seipens have important roles. In legumes, suitable experimental systems are required that can point to the most appropriate genes for stacking, for subsequent whole plant analysis. Such systems are available in the model legume *M. truncatula*, using transient leaf expression and somatic embryos (Picard et al. 2013; Song et al. 2013).

Regulation of carbon partitioning determines the mix of carbohydrate (sugars, starch, cell wall storage polysaccharides) and oil. Legumes with their diversity of seed protein, oil and carbohydrate offer potential for progressing understanding of the key determinants of storage partitioning. The large amount of variation in the legume family is a major resource for the future (Gresshoff et al. 2015). Improved understanding of partitioning will likely require gene knockdown to reduce carbon flow into some pathways, to promote oil biosynthesis. Knockdown can be facilitated by the utilization of CRISR-cas9 technology (Luo et al. 2016).

There are other opportunities with non-transgenic molecular breeding approaches (Chaudhary et al. 2015). The improved knowledge of oil body biogenesis, particularly in relation to oil body assembly, together with available genomic resources can facilitate development of selection markers. The current knowledge of biochemistry and molecular biology knowledge can be linked to the information derived from QTLs (Quantitative Trait Loci) and genome -wide association studies, available in legume crops such as soybean (Chaudhary et al. 2015). Meta-QTL analysis has been carried out on oil content in legumes (Qi et al. 2011; Zhaoming et al. 2017). Identification of transcript polymorphisms using integrated RNA-seq and bioinformatics is another approach to identify lines varying in oil composition (Goettel et al. 2014). CRISPR-cas9 gene-editing technology can be utilized where there are useful variants of known genes and regulatory regions (Luo et al. 2016).

Biotechnology to enhance oil production in legumes can be utilized not only in oil crops but for nutritional enhancement in other food legumes with low-lipid seed stores. While maximizing oil levels is a major biotechnological objective, there is also substantive potential in modifying fatty acids for different types of oil utilization as well as using oil bodies as a vehicle for seed protein enrichment.

### **Author contribution statement**

YS and RJR designed and wrote the review; X-D Wang did the oil body cytology.
